# [Retracted] Fbxw7 regulates hepatocellular carcinoma migration and invasion via Notch1 signaling pathway

**DOI:** 10.3892/ijo.2025.5763

**Published:** 2025-06-16

**Authors:** Xing Wang, Juan Zhang, Liang Zhou, Wei Sun, Zhi-Gang Zheng, Peng Lu, Yuan Gao, Xi-Sheng Yang, Zhuo-Chao Zhang, Kai-Shan Tao, Ke-Feng Dou

Int J Oncol 47: 231-243, 2015; DOI: 10.3892/ijo.2015.2981

Following the publication of this paper, it was drawn to the Editor's attention by a concerned reader that, for the different cell invasion and migration assay experiments shown in Fig. 5 on p. 238, as many as four pairs of overlapping data panels were identified, such that these data panels had all apparently been derived from the same original source(s); in addition, a further pair of overlapping data panels was identified comparing between Fig. 5 and the cell migration and invasion assay experiments shown in Fig. 7.

Owing to the large number of duplications of data that have been identified in this paper, the Editor of *International Journal of Oncology* has decided that it should be retracted from the Journal on account of a lack of confidence in the presented data. The authors were asked for an explanation to account for these concerns, but the Editorial Office did not receive a satisfactory reply. The Editor apologizes to the readership for any inconvenience caused.

